# MLST-based inference of genetic diversity and population structure of clinical *Klebsiella pneumoniae*, China

**DOI:** 10.1038/srep07612

**Published:** 2015-01-05

**Authors:** Chenyi Guo, Xianwei Yang, Yarong Wu, Huiying Yang, Yanping Han, Ruifu Yang, Liangping Hu, Yujun Cui, Dongsheng Zhou

**Affiliations:** 1 Consulting Center of Biomedical Statistics, Beijing 100850, China; 2 State Key Laboratory of Pathogen and Biosecurity, Beijing Institute of Microbiology and Epidemiology, Beijing 100071, China

## Abstract

Multilocus sequence typing was applied to a collection of 327 clinical isolates of *Klebsiella pneumoniae* from China, which was proven to be a good representative of the global diversity of *K. pneumoniae*. Three lineages L1 to L3 are presented in the population with limited genetic flow across different lineages. However, extremely high levels of recombination can be observed within lineages to the extent at which the alleles are associated almost randomly. Lineages L2 and L3 most likely represent highly specific subgroups of less-virulent *K. pneumoniae* with modified metabolic networks, while lineage L1 contains not only hypervirulent clones with massive acquisition of virulent genes but also ‘primitive and intermediate forms’ during evolution of hypervirulent *K. pneumoniae*.

K*lebsiella pneumoniae* commonly causes nosocomial infections in urinary tract, respiratory tract and blood; under these circumstances, this bacterium is considered as an opportunistic pathogen since it mostly affects debilitated patients^1^. Nosocomial isolates of *K. pneumoniae* often display drug resistance phenotypes, making difficulty in choosing sensitive antibiotics for treatment^2^,^3^. In addition, a subset of capsular serotypes (including predominantly K1 and K2) constitute hypervirulent variants of *K. pneumoniae* that have emerged worldwide in the past two decades^4^,^5^,^6^. With increased production of the major virulence determinant capsular polysaccharide, these hypervirulent variants affect previously healthy persons to cause often community-acquired, life-threatening infections such as pyogenic liver abscess, meningitis, and necrotizing fasciitis^4^,^5^,^6^.

Genotyping is important to identify cases or outbreaks due to *K. pneumoniae* and to further track source and spreading of infections. The major genotyping methods of *K. pneumoniae* include pulsed field gel electrophoresis (PFGE), multiple-locus variable number tandem repeat analysis (MLVA) and multilocus sequence typing (MLST)^7^,^8^, and among them MLST is the most popular one. The *K. pneumoniae* MLST scheme was developed in 2005^8^ and then used globally to characterize diversity and epidemiology of clinical *K. pneumoniae* isolates, leading to identification of various clones that differ sharply by their features of virulence or drug resistance^9^,^10^,^11^,^12^,^13^,^14^,^15^.

In our previous study, a genotyping scheme based on the prevalence of 41 large variably-presented gene clusters (LVPCs; four of them correspond to four different virulence loci) plus seven additional virulence markers was established with a collection of 327 clinical isolates of from China, which could be grouped into eight genetically distinct complexes^16^. *K. pneumoniae* strains have horizontally acquired various genomic loci including those contributing to virulence during evolution of ‘classic’ opportunistic forms into hypervirulent variants^16^. In this follow-up study, a modified MLST scheme was established and applied to the same strain collection, providing an extended dissection of population genetics, phylogeny and epidemiology of *K. pneumoniae*.

## Results and Discussion

### Extended MLST scheme

The existent *K. pneumoniae* MLST scheme^8^ employs seven loci *gapA*, *infB*, *mdh*, *pgi*, *phoE*, *recA* and *tonB*, and a total of 1595 STs have been deposited in the *K. pneumoniae* MLST Database (http://www.pasteur.fr/recherche/genopole/PF8/mlst/Kpneumoniae.html, last accessed March 20, 2014). When we applied this MLST scheme to our strain collection, redesign of primers (but retaining the locations of allele sequence) brought greatly enhanced amplification success rates for the former six loci; however, repeated attempts with different PCR conditions and primers still led to poor amplification performance for *tonB*, which was ultimately replaced by the *rpoB* (beta-subunit of RNA polymerase) gene (Table S1). Poor amplification performance of *tonB* might be due to frequent insertion/deletion events of one or more codons in the primer annealing regions^10^. Based on the shared six loci *gapA*, *infB*, *mdh*, *pgi*, *phoE*, and *recA*, we built a NJ tree involving the 1474 STs from the above MLST database plus the 128 STs (see below) from the 327 isolates tested in this study (Figure S1). The uniform scatter of the 128 STs in the NJ tree indicated that our strain collection was a good representative of the global genetic diversity of *K. pneumoniae*.

### Sequence diversity under purifying selection

Sequence alignment of each of the seven loci showed no insertion/deletion, and the concatenated sequence for the seven loci was 2,945 bp in length. There were 273 (9.27%) polymorphic sites detected in total, of which 21 were tri-allelic SNPs (Table 1). The number of alleles found at each DNA fragment ranged from 21 (*gapA*) to 46 (*phoE*). The diversity index π was 0.01086 for the concatenated sequences and ranged from 0.0046 (*gapA*) to 0.0175 (*recA*) at different loci.

*d*_N_/*d*_S_ > 1 or <1 indicated positive or negative selection on the gene sequence tested, respectively. The *d_N_/d_S_* ratios for the seven loci varied from 0.00 (*recA*) to 0.139 (*phoE*), and that for the concatenated sequence was 0.047, indicating strong purifying selection on these genes.

### STs and CCs

**Figure 1:**
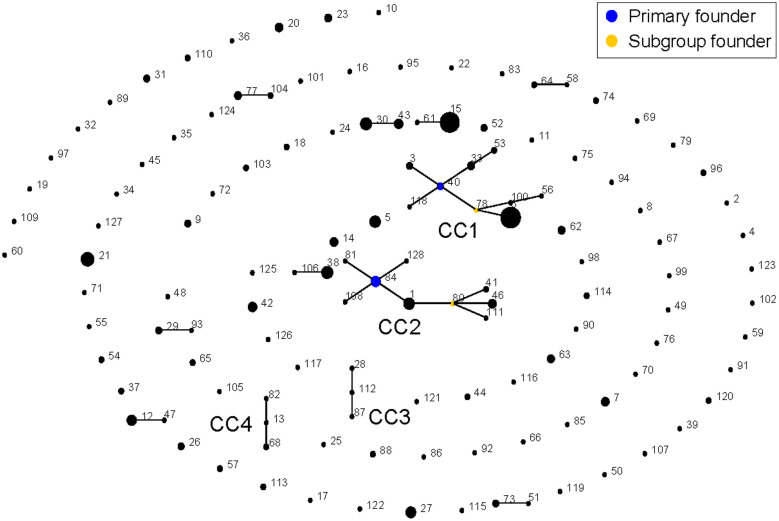
Population snapshot. The population snapshot of the 327 isolates was diagramed by eBURST on the basis of allelic profiles. STs with SLV relationship were linked together to form 4 CCs (CC1 to CC4) and 8 doubletons. The size of the circle represented the number of isolates of each ST.

A total of 128 unique STs were identified from the 327 isolates tested, which were assigned into 4 CCs (CC1 to CC4; 82 strains), 8 doubletons (82 strains) and 88 singletons (163) (Figure 1). CC1 to CC4 contained 9 (47 strains), 9 (28), 3 (3), and 3 (4) STs, respectively. Usually, the predicted founder corresponds to the most predominant ST in a CC^17^. However, the CC1 founder ST40 contained only three isolates (6.4% of the total 47 strains in CC1), while its DLV descendant ST6 was composed of 31 isolates. Similarly, the CC2 founder ST84 was also not the predominant ST in the complex. This might be resulted from the sampling bias, or due to the reason that the founder ST was swept by selection pressure such as wide application of specific antibiotics in clinic.

### Three lineages in the whole population

**Figure 2:**
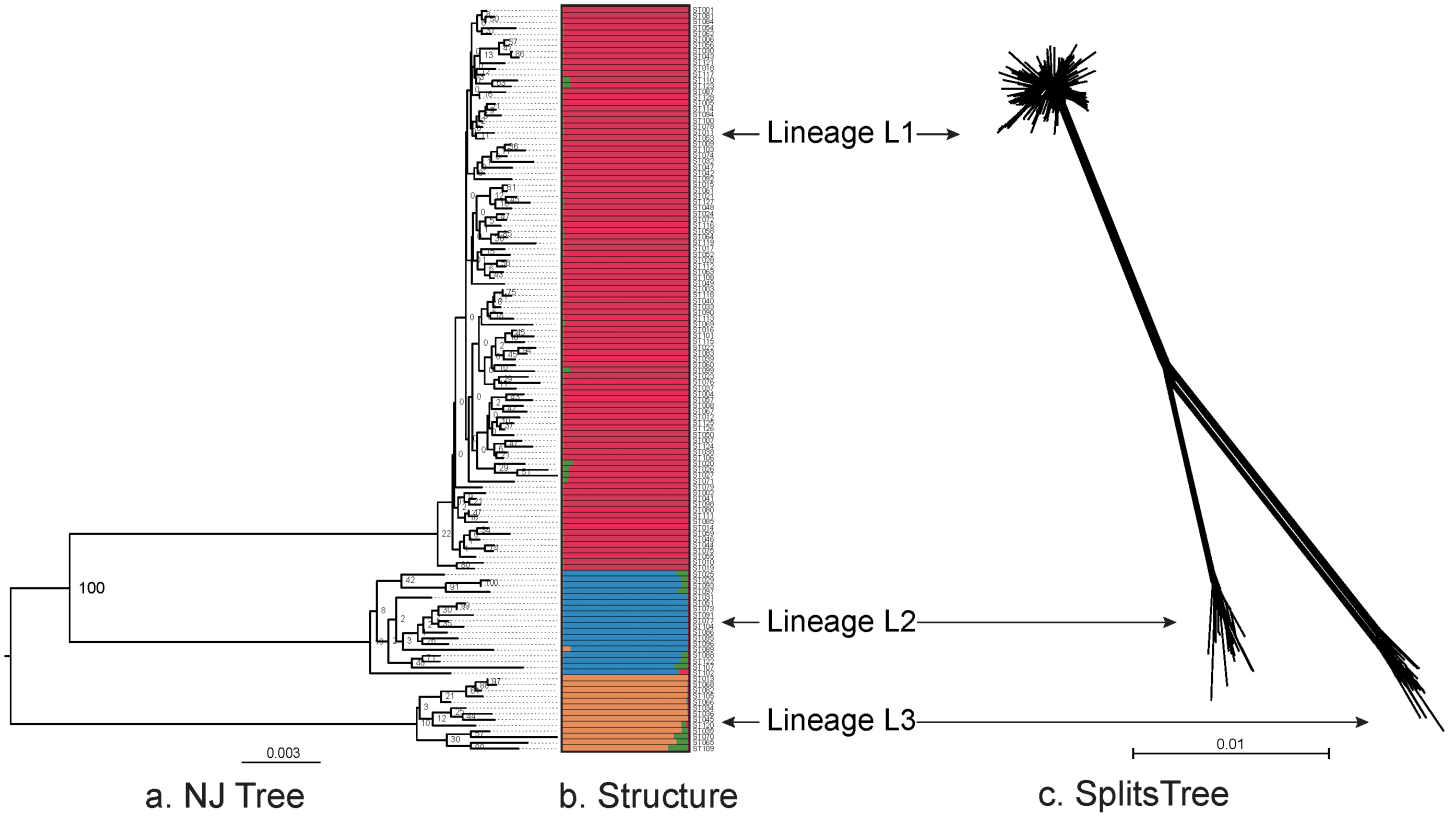
Diagrams denoting population structure. (a). Unrooted NJ tree of the 128 STs based on the concatenated sequences of 7 loci and the three lineages (L1 to L3) were separated with 100% bootstrap value support. (b). Proportions of ancestral subpopulations of the 128 STs and different colors represented distinct assuming subpopulations corresponding to lineage L1 to L3. (c). Splites network of the 128 STs generated by neighbor-net method using SplitsTree4 based on the concatenated sequences of 7 loci. The three subgroups corresponding to lineage L1 to L3 could be discriminated.

A NJ tree was built from the concatenated sequences of the 128 STs (Figure 2a). Three distinct lineages, termed L1 to L3, were observed with 100% of bootstrap supporting. Remarkably, the bootstrap values on the branches within all the three lineages were extremely low even to zero, suggesting frequent homologous recombination occurred across these branches and eradicated phylogenetic signals of vertical inheritance.

The linkage model of STRUCTURE was applied to the sequence dataset of 128 STs. Multiple runs with *K* values from 2 to 15 showed maximal posterior probability at *K* = 4. The 128 STs fell into three distinct subgroups (corresponding to lineage L1 to L3) according to the major ancestral population designation of each ST (Figure 2B). There were little admixture of ancestral sources between these three subgroups, and STs within each subgroup tended to be highly homogenous. In addition, the splits network of the 128 STs also revealed three distinct subgroups corresponding to lineage L1 to L3 (Figure 2c). An overall bifurcating structure was observed from the three lineages with less visualized intersections across different lineages, but each lineage displayed a very complex interconnecting network structure. The above observations further confirmed limited and frequent gene flow across and within lineages, respectively.

In addition, three corresponding major lineages could also be found in the NJ tree of the 128 + 1474 STs (Figure S1). Therefore, the three major lineages would reflect the basic population structure feature of *K. pneumoniae* of global origins.

### Extremely frequent gene flow within lineages

The *P* value determined by the *phi* test for the 128 STs (whole population) and those for the ST collections in different lineages were all <0.001, indicating recombination events occurred within and across lineages (Table 2). This result agreed with visualized inspections across and within lineages as determined by SplitsTree (Figure 2c). The detecting per-site ρ/θ value for the 128 STs was 0.42, suggesting point mutation was 2.38 times more likely to occur than recombination at the level of whole population. However, the ρ/θ ratio values were 30.79, 31.94 and 13.12 for lineage L1 to L3, respectively. The recombination frequency within lineages was at least 31 times higher than that across different lineages.

The st. *I_A_* values were 0.0107 (*P* = 0.142), 0.0424 (*P* = 0.118) and 0.0507 (*P* = 0.0741) for lineage L1 to L3, respectively, suggesting a tendency of free recombination between the alleles in each lineage. By contrast, the st. *I_A_* of the 128 STs was 0.1644 (*P* < 0.0001), which was significantly different from zero, indicating a tendency of linkage disequilibrium between the alleles at the level of whole population.

Taken the above together, recombination was highly frequent within lineages but limited across lineages, suggesting natural barriers were presented to prevent gene flow across lineages. Isolates from each sampling city or year could be found in all the three lineages (Figure S2), displaying no evident lineage-specific distribution of isolates with respect to time and geography. Therefore, the natural barriers between lineages might result from high levels of DNA sequence mismatch between donor and recipient^18^.

### Nonsense mutations in *pgi*

Four kinds of nonsense mutation in *pgi* were unexpectedly identified from 32 STs (52 isolates), and occurred due to substitution from codon TGG to TGA at nucleotide positions 117, 183, 186 and 216, respectively. The *pgi* gene encodes the phosphoglucose isomerase, which catalyzes isomerization of glucose 6-phosphate to fructose 6-phosphate in upper glycolysis^19^. Notably, *E. coli* lacking *pgi* remains viable and the loss of *pgi* forces glycolytic flux through the pentose phosphate pathway, creating a redox imbalance due to excess NADPH production^19^. Interestingly, *pgi* nonsense mutations could be found in all the 18 and 13 STs from lineages L2 and L3, respectively. By contrast, only 5 of the 97 STs in L1 presented *pgi* nonsense mutations. It was speculated that *pgi* nonsense mutations might have a positive effect on relevant phenotypes, increasing the fitness of the L2 and L3 organisms in specific niches.

### Comparison to previous LVPC-based genotyping

**Figure 3:**
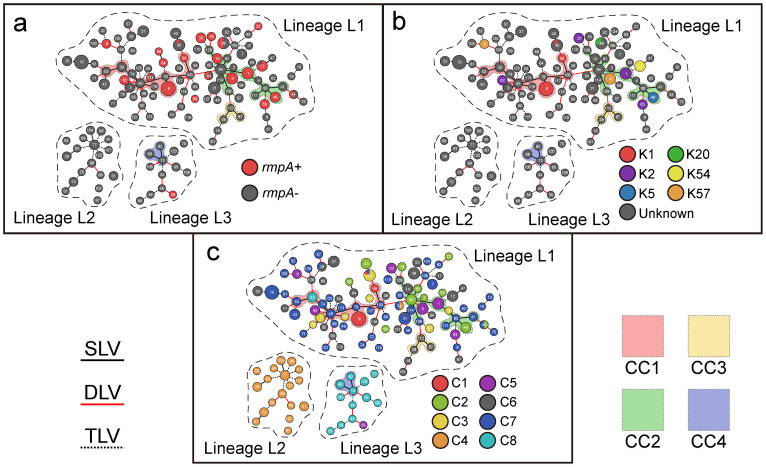
MS tree. The MS tree of the 128 STs was inferred by Phyloviz on the basis of allelic profiles. Each circle indicated a ST (node), and a larger size of the circle corresponded to a larger number of strains included. The edge between STs represented the relationships of SLV (black and solid), DLV (red and solid) and TLV (black and dash), and a thicker edge corresponded to a shorter phylogenic distance. The dash lines separated three lineages L1 to L3. The background colors indicated different CCs. The prevalence of *rmpA* (a), capcular serotypes K1, K2, K5, K20, K54, K57 (b) and LVPC complexes C1 to C8 (c) were highlighted with different colors.

The goeBurst analysis of the allelic profiles of all the 327 strains generated a minimum spanning (MS) tree to provide an intuitive view of the phylogenetic relationships between STs, singletons, doubletons, CCs and lineages (Figure 3). Allelic profile-based phylogenetic relationships as inferred from categorical codes were more reliable than nucleotide-based phylogenies, because replacement of an allele by recombination is scored as a single event^20^,^21^. As expected, the structure of the three detected lineages could be illustrated and CC1 to CC3 were found in lineage L1 while CC in L3.

The prevalence of *rmpA* (Figure 3a), capsular serotypes K1, K2, K5, K20, K54 and K57 (Figure 3b) and LVPC-based complexes C1 to C8 (Figure 3c), as characterized previously^16^, was highlighted in the MS tree. The *rmpA* gene, which encodes a positive regulator of capsular polysaccharide biosynthesis, is closely associated with the hypervirulent phenotype^7^,^22^,^23^. Twenty-two (99 isolates) of the 128 STs carried *rmpA*. Except for ST35 (one isolate), all the other *rmpA*-positive STs belonged to lineage L1. Notably, isolates within each of ST12, ST30, ST38, ST44, ST54 and ST113 may be either *rmpA*-positive or *rmpA*-negative. As *rmpA* was dispersed in different STs and CCs, the spread of this gene in the population might be due to separated events of horizontal gene transfer rather than vertical transmission from a common ancestor.

Except one strain of K54, all the K1, K2, K5, K20, K54, and K57 strains were *rmpA*-positive, indicating that most of them were closely related to the hypervirulent phenotype^16^. All these isolates with available serotypes belonged to lineage L1. K1 corresponded to three genetically closed STs (ST6, ST56 and ST30); the former two belonged to CC1 while the last one ST30 differed from ST56 by two alleles. K2 corresponded to ST62, ST63, ST20 and ST1; the former three were singletons while ST1 belonged to CC2. K57 was found in the two singletons ST5 and ST9. K5 were found in ST46 of CC2. K20 or K54 was found in a single singleton ST38 or ST12. The above results were consistent with the previous MLST-based notion that serotypes were not strongly associated with genotype background^10^.

Lineage L2 exclusively included 30 of the 31 isolates from LVPC-based complex C4, and all STs except for ST35 in L3 corresponded to 15 of the 19 strains from C8. The remaining one and four isolates from C4 and C8, respectively, were attributed to lineage L1. All the L2 and L3 strains were *rmpA*-negative and moreover C4 and C8 had been characterized as subgroups of less-virulent *K. pneumoniae* with very limited acquisition of virulent gene loci^16^, and thus lineages L2 and L3 were mostly like closely related to less-virulent *K. pneumoniae*. Except for one isolate (ST35) from C5, all isolates of C1, C2, C3, C5, C6 and C7 were included in lineage L1. Lineage L1 appeared to a very complex mixture including not only hypervirulent clones but also ‘primitive and intermediate forms’ during evolution of hypervirulent *K. pneumoniae*.

### Concluding remarks

This is the first report of MLST-based inference of genetic diversity and population structure of clinical *K. pneumoniae* isolated from China. Notably, our strain collection is a good representative of the global diversity of clinical *K. pneumoniae*. At least three major lineages L1 to L3 are presented in the *K. pneumoniae* population with limited horizontal exchange of genetic materials across lineages. However, there are extremely high levels of recombination within lineages to the extent at which the alleles are associated almost randomly (i.e. a tendency to linkage equilibrium). Lineages L2 and L3 most likely represent highly specific subgroups of less-virulent *K. pneumoniae* with modified metabolic networks, while lineage L1 contains not only hypervirulent clones with massive acquisition of virulent genes but also ‘primitive and intermediate forms’ during evolution of hypervirulent *K. pneumoniae*. Further genome sequencing study on a large collection of representative clinical isolates of *K. pneumoniae* will give a much deeper understanding of genetic diversity, phylogeny, population structure and epidemiology of this pathogen.

## Methods

### Bacterial strains

A total of 327 clinical isolates of *K. pneumoniae* were tested in this work, all of which were involved in our previous LVPC-based genotyping study^16^. Beside the reference strain NTUH-K2044 with determined genome sequence^24^, the remaining 326 strains, being isolated between 2004 and 2009, came from the hospitals in Beijing (North China), Chongqing (Southwest China) and Shenzhen (South China). Genomic DNAs were isolated by classical phenol/chloroform method followed by methoxyethanol removal of polysaccharides that contaminate genomic DNA^25^, and then arrayed in 96-well PCR plates for further analyses.

### PCR amplification and sequencing

PCR primers (Table S2) of target genes were designed with NTUH-K2044 sequences. A volume of 50 μl PCR mixture contained 50 mM KCl, 10 mM Tris-HCl (pH8.0), 2.5 mM MgCl2, 0.001% gelatin, 0.1% BSA, 100 μM of each dATP, dCTP, dGTP and dTTP, 0.1 μM of each primer, 1 unit of each of ExTaq polymerases (TaKaRa), and 10 ng of genomic DNA. The amplification conditions were as follows: 95°C for 5 min, and then 30 cycles of 94°C for 40 s, an appropriate annealing temperature (Table S2) for 40 s, and 72°C for 1 min. PCR products were analyzed by agarose gel electrophoresis and purified by ultrafiltration (Millipore). Both DNA strands were sequenced with PCR primers on ABI-3700 sequencer. DNA sequences were aligned using MUSCLE Version 3.8^26^.

### Sequence diversity analyses

The G + C content, number of polymorphic sites, average pairwise nucleotide, and difference per site (π) were calculated with DnaSP Version 5.10^27^. The average non-synonymous/synonymous rate ratio (*d_N_/d_S_*) was calculated with KaKs Calculator Version 2.0^28^ to infer direction and magnitude of natural selection.

### Allelic diversity analyses

DNA sequences of each of the seven MLST loci that differed from each other by one or more polymorphisms were assigned with different allele numbers. Distinct allelic profiles were assigned with different sequence types (STs). Clustering of related STs was carried out by eBURST Version 3^17^. Two different STs sharing six of the seven loci constituted a single-locus variant (SLV). A double-locus variant (DLV) contained two STs differing in two loci and other loci should be identical. A triple-locus variant (TLV) included two STs differing in three loci. A clonal complex was composed of at least three STs with only SLVs. Only two STs belong to the same group with SLV was called doublet. The remaining STs, which had no SLV with other STs, were termed singletons. The founders (ancestry types) of CCs were predicted with 1,000 re-samplings for bootstrap.

### Population structure analyses

The Neighbor Joining (NJ) method^29^ was used to build phylogenetic trees of strains or STs. STRUCTURE software Version 2.3^30^,^31^,^32^,^33^ was used with linkage model to infer ancestry of STs, and this procedure assumed that each ST was derived from *K* assuming ancestral subpopulations. The proportions for each ST of *K* subpopulations could be estimated and illustrated. The posterior probability P(X|K) was calculated to determine which *K* to choose, where *X* stood for the number of genotypes of sampled isolates. 14 individual runs (20,000 burn-in iterations and 30,000 iterations sampling iterations) per value of *K* ranged from 2 to 15 were performed and 4 was chosen as the appropriate ancestry number with maximal posterior probability. The splits network of STs was generated by neighbor-net method^34^ using SplitsTree4^35^. Global optimal eBURST implemented by Phyloviz^36^ was used to cluster STs with triple-locus variant (TLV) limitation, generating a MS tree to visualize possible evolutionary relationships between STs.

### Recombination analyses

The *phi* test for recombination was performed with SplitsTree4^35^, and *P* values < 0.05 indicated recombination existed. The Linkage Analysis Version 3.6^37^ was used to calculate standardized index of association (st. *I_A_*) with 10,000 iterations by Monte Carlo based on allelic profiles. If there was linkage equilibrium because of frequent recombination events, the expected value of st. *I_A_* was zero, which suggested no association between alleles at different loci; if st. *I_A_* was statistically significant different from zero, alleles were suggested with genetic linkage. The LDhat program^38^,^39^ implemented in the RDP4 package^40^ was used to calculate per-site ρ/θ ratios based on concatenated sequences of the seven loci with 1,000,000 MCMC updates. The parameters ρ and θ represented the rates of recombination and mutation respectively.

## Supplementary Material

Supplementary Information 3 Supplementary material V2

## Figures and Tables

**Table 1:** Nucleotide and allelic sequence diversity

Locus	Length (bp)	No. of alleles	Average G + C content (%)	No. of SNPs	No. of polymorphic sites (%)	Average *d*N/*d*S ratio	No. of nonsense mutation	π
*gapA*	450	21	56.19	25	25 (5.56)	0.028	-	0.004562
*infB*	318	22	62.37	26	29 (9.12)	0.01	-	0.010844
*mdh*	477	27	56.67	47	51 (10.69)	0.054	-	0.014029
*pgi*	432	29	58.41	43	51 (11.81)	0.042	4	0.013856
*phoE*	420	46	55.80	48	51 (12.14)	0.139	-	0.009281
*recA*	347	33	61.40	27	29 (8.36)	0	-	0.017538
*rpoB*	501	24	54.98	36	37 (7.39)	0.036	-	0.007624
Concatenated	2945	128	57.61	252	273 (9.27)	0.047	4	0.01086

**Table 2:** Recombination test and estimation

		Recombination					Linkage disequilibrium	
Population (n)	*phi*	theta/site	rho/site	LB 95%	UP 95%	rho/theta	I_A_	*P* value
Whole (128)	<0.001	1.57E-02	6.75E-03	5.23E-03	8.57E-03	0.42	0.1644	<0.0001
L1 (97)	<0.001	6.55E-03	2.02E-01	1.04E-01	4.25E-01	30.79	0.0107	0.142
L2 (18)	<0.001	6.23E-03	1.99E-01	5.48E-02	1.31E+00	31.94	0.0351	0.138
L3 (13)	<0.001	6.09E-03	7.99E-02	5.68E-02	1.12E-01	13.12	0.0507	0.0741

## References

[b1] Podschun R. & Ullmann U. *Klebsiella* spp. as nosocomial pathogens: epidemiology, taxonomy, typing methods, and pathogenicity factors. Clin. Microbiol. Rev. 11, 589–603 (1998). 9767057 10.1128/cmr.11.4.589 PMC88898

[b2] Paterson D. L. *et al.* International prospective study of *Klebsiella pneumoniae* bacteremia: implications of extended-spectrum beta-lactamase production in nosocomial Infections. Ann. Intern. Med. 140, 26–32 (2004). 14706969 10.7326/0003-4819-140-1-200401060-00008

[b3] Keynan Y. & Rubinstein E. The changing face of *Klebsiella pneumoniae* infections in the community. Int. J. Antimicrob. Agents 30, 385–389 (2007). 17716872 10.1016/j.ijantimicag.2007.06.019

[b4] Siu L. K., Yeh K. M., Lin J. C., Fung C. P. & Chang F. Y. *Klebsiella pneumoniae* liver abscess: a new invasive syndrome. Lancet Infect. Dis. 12, 881–887 (2012). 23099082 10.1016/S1473-3099(12)70205-0

[b5] Shon A. S. & Russo T. A. Hypervirulent *Klebsiella pneumoniae*: the next superbug? Future Microbiol. 7, 669–671 (2012). 22702521 10.2217/fmb.12.43

[b6] Shon A. S., Bajwa R. P. & Russo T. A. Hypervirulent (hypermucoviscous) *Klebsiella pneumoniae*: a new and dangerous breed. Virulence 4, 107–118, 10.4161/viru.22718 (2013). 23302790 PMC3654609

[b7] Turton J. F., Perry C., Elgohari S. & Hampton C. V. PCR characterization and typing of *Klebsiella pneumoniae* using capsular type-specific, variable number tandem repeat and virulence gene targets. J. Med. Microbiol. 59, 541–547 (2010). 20110386 10.1099/jmm.0.015198-0

[b8] Diancourt L., Passet V., Verhoef J., Grimont P. A. & Brisse S. Multilocus sequence typing of *Klebsiella pneumoniae* nosocomial isolates. J. Clin. Microbiol. 43, 4178–4182 (2005). 16081970 10.1128/JCM.43.8.4178-4182.2005 PMC1233940

[b9] Wang Q. *et al.* Genotypic Analysis of *Klebsiella pneumoniae* Isolates in a Beijing Hospital Reveals High Genetic Diversity and Clonal Population Structure of Drug-Resistant Isolates. PLoS One 8, e57091, 10.1371/journal.pone.0057091 (2013). 23437318 PMC3578803

[b10] Brisse S. *et al.* Virulent clones of *Klebsiella pneumoniae*: identification and evolutionary scenario based on genomic and phenotypic characterization. PLoS One 4, e4982, 10.1371/journal.pone.0004982 (2009). 19319196 PMC2656620

[b11] Harada S. *et al.* Familial spread of a virulent clone of *Klebsiella pneumoniae* causing primary liver abscess. J. Clin. Microbiol. 49, 2354–2356 (2011). 21490191 10.1128/JCM.00034-11 PMC3122714

[b12] Siu L. K. *et al.* Molecular typing and virulence analysis of serotype K1 *Klebsiella pneumoniae* strains isolated from liver abscess patients and stool samples from noninfectious subjects in Hong Kong, Singapore, and Taiwan. J. Clin. Microbiol. 49, 3761–3765 (2011). 21900521 10.1128/JCM.00977-11 PMC3209116

[b13] Lin J. C. *et al.* Genotypes and virulence in serotype K2 *Klebsiella pneumoniae* from liver abscess and non-infectious carriers in Hong Kong, Singapore and Taiwan. Gut. Pathog. 6, 21, 10.1186/1757-4749-6-21 (2014). 24987462 PMC4076766

[b14] Luo Y., Wang Y., Ye L. & Yang J. Molecular epidemiology and virulence factors of pyogenic liver abscess causing *Klebsiella pneumoniae* in China. Clin. Microbiol. Infect., 10.1111/1469-0691.12664 (2014). 24804560

[b15] Chen L., Mathema B., Pitout J. D., DeLeo F. R. & Kreiswirth B. N. Epidemic *Klebsiella pneumoniae* ST258 is a hybrid strain. MBio 5, e01355–14, 10.1128/mBio.01355-14 (2014). 24961694 PMC4073492

[b16] Chen Z. *et al.* A novel PCR-based genotyping scheme for clinical *Klebsiella pneumoniae*. Future Microbiol. 9, 21–32 (2014). 24328378 10.2217/fmb.13.137

[b17] Feil E. J., Li B. C., Aanensen D. M., Hanage W. P. & Spratt B. G. eBURST: inferring patterns of evolutionary descent among clusters of related bacterial genotypes from multilocus sequence typing data. J. Bacteriol. 186, 1518–1530 (2004). 14973027 10.1128/JB.186.5.1518-1530.2004 PMC344416

[b18] Matic I., Radman M. & Rayssiguier C. Structure of recombinants from conjugational crosses between *Escherichia coli* donor and mismatch-repair deficient *Salmonella typhimurium* recipients. Genetics 136, 17–26 (1994). 8138154 10.1093/genetics/136.1.17 PMC1205768

[b19] Charusanti P. *et al.* Genetic basis of growth adaptation of *Escherichia coli* after deletion of *pgi*, a major metabolic gene. PLoS Genet. 6, e1001186, 10.1371/journal.pgen.1001186 (2010). 21079674 PMC2973815

[b20] Spratt B. G., Hanage W. P. & Feil E. J. The relative contributions of recombination and point mutation to the diversification of bacterial clones. Curr. Opin. Microbiol. 4, 602–606 (2001). 11587939 10.1016/s1369-5274(00)00257-5

[b21] Salerno A. *et al.* Recombining population structure of *Plesiomonas shigelloides* (Enterobacteriaceae) revealed by multilocus sequence typing. J. Bacteriol. 189, 7808–7818 (2007). 17693512 10.1128/JB.00796-07 PMC2168737

[b22] Yu W. L. *et al.* Association between *rmpA* and *magA* genes and clinical syndromes caused by *Klebsiella pneumoniae* in Taiwan. Clin. Infect. Dis. 42, 1351–1358 (2006). 16619144 10.1086/503420

[b23] Hsu C. R., Lin T. L., Chen Y. C., Chou H. C. & Wang J. T. The role of *Klebsiella pneumoniae rmpA* in capsular polysaccharide synthesis and virulence revisited. Microbiology, 157, 3446–3457 (2011). 21964731 10.1099/mic.0.050336-0

[b24] Wu K. M. *et al.* Genome sequencing and comparative analysis of *Klebsiella pneumoniae* NTUH-K2044, a strain causing liver abscess and meningitis. J. Bacteriol. 191, 4492–4501 (2009). 19447910 10.1128/JB.00315-09 PMC2704730

[b25] Xiao X. *et al.* Two methods for extraction of high-purity genomic DNA from mucoid Gram-negative bacteria. Afric. J. Microbiol. Res. 5, 4013–4018 (2011).

[b26] Edgar R. C. MUSCLE: multiple sequence alignment with high accuracy and high throughput. Nucleic Acids Res. 32, 1792–1797 (2004). 15034147 10.1093/nar/gkh340 PMC390337

[b27] Librado P. & Rozas J. DnaSP v5: a software for comprehensive analysis of DNA polymorphism data. Bioinformatics 25, 1451–1452 (2009). 19346325 10.1093/bioinformatics/btp187

[b28] Wang D., Zhang Y., Zhang Z., Zhu J. & Yu J. KaKs_Calculator 2.0: a toolkit incorporating gamma-series methods and sliding window strategies. Genomics, Proteomics & Bioinformatics 8, 77–80 (2010). 10.1016/S1672-0229(10)60008-3 PMC5054116 20451164

[b29] Saitou N. & Nei M. The neighbor-joining method: a new method for reconstructing phylogenetic trees. Mol. Biol. Evol. 4, 406–425 (1987). 3447015 10.1093/oxfordjournals.molbev.a040454

[b30] Pritchard J. K., Stephens M. & Donnelly P. Inference of population structure using multilocus genotype data. Genetics 155, 945–959 (2000). 10835412 10.1093/genetics/155.2.945 PMC1461096

[b31] Falush D., Stephens M. & Pritchard J. K. Inference of population structure using multilocus genotype data: linked loci and correlated allele frequencies. Genetics 164, 1567–1587 (2003). 12930761 10.1093/genetics/164.4.1567 PMC1462648

[b32] Falush D., Stephens M. & Pritchard J. K. Inference of population structure using multilocus genotype data: dominant markers and null alleles. Mol. Ecol. Notes 7, 574–578 (2007). 18784791 10.1111/j.1471-8286.2007.01758.x PMC1974779

[b33] Hubisz M. J., Falush D., Stephens M. & Pritchard J. K. Inferring weak population structure with the assistance of sample group information. Mol. Ecol. Resour. 9, 1322–1332 (2009). 21564903 10.1111/j.1755-0998.2009.02591.x PMC3518025

[b34] Bryant D. & Moulton V. Neighbor-Net: an agglomerative method for the construction of phylogenetic networks. in Algorithms in Bioinformatics, 375–391 (Springer, 2002). 10.1093/molbev/msh018 14660700

[b35] Bruen T. C., Philippe H. & Bryant D. A simple and robust statistical test for detecting the presence of recombination. Genetics 172, 2665–2681 (2006). 16489234 10.1534/genetics.105.048975 PMC1456386

[b36] Francisco A. P. *et al.* PHYLOViZ: phylogenetic inference and data visualization for sequence based typing methods. BMC Bioinformatics 13, 87 (2012). 22568821 10.1186/1471-2105-13-87 PMC3403920

[b37] Haubold B. & Hudson R. R. LIAN 3.0: detecting linkage disequilibrium in multilocus data. Linkage Analysis. Bioinformatics 16, 847–848 (2000). 11108709 10.1093/bioinformatics/16.9.847

[b38] McVean G. A. *et al.* The fine-scale structure of recombination rate variation in the human genome. Science 304, 581–584 (2004). 15105499 10.1126/science.1092500

[b39] Auton A. & McVean G. Recombination rate estimation in the presence of hotspots. Genome Res. 17, 1219–1227 (2007). 17623807 10.1101/gr.6386707 PMC1933511

[b40] Martin D. P. *et al.* RDP3: a flexible and fast computer program for analyzing recombination. Bioinformatics 26, 2462–2463 (2010). 20798170 10.1093/bioinformatics/btq467 PMC2944210

