# Supplementary material for: MLST-based inference of genetic diversity and population structure of clinical Klebsiella pneumoniae, China
Source: Sci Rep. 2015 Jan 5;5:7612. doi: 10.1038/srep07612 (PMC5154592; doi:10.1038/srep07612)
Supplement: Supplementary Information — 3 Supplementary material V2 [file srep07612-s1.pdf]

Supplementary Information

MLST-based inference of genetic diversity and population structure of clinical *Klebsiella pneumoniae*, China

Chenyi Guo[1], Xianwei Yang[2], Yarong Wu[2], Huiying Yang[2], Yanping Han[2], Ruifu Yang[2], Liangping Hu[1], Yujun Cui[2], Dongsheng Zhou[2]

1. Consulting Center of Biomedical Statistics, Beijing 100850, China; 2. State Key Laboratory of Pathogen and Biosecurity, Beijing Institute of Microbiology and Epidemiology, Beijing 100071, China

Table S1. Primers used in this study

Locus	Length (bp)	Primers	Annealing temperature
gapA	450	KP-MLST-gapA-F: CCACTCACGGTCGTTTCG<br>KP-MLST-gapA-R: ATGGCTTTCTTGATTTCTTC	56℃
infB	318	KP-MLST-infB-F: GACTACATTCGTTCTACCAAGG<br>KP-MLST-infB-R: CAAGGAAGGATTCGATGAC	56℃
mdh	477	KP-MLST-mdh-F1: CGTTGTACGACATCGCTCC<br>KP-MLST-mdh-R1: CGGCTGGGAGAAGAAACG	59℃
pgi	432	KP-MLST-pgi-F: TACAACAACTTCTTCGGTGC<br>KP-MLST-pgi-R: AGGATGCGGTTAGCCAGC	59℃
phoE	420	KP-MLST-phoE-F: TCTTCGGTCTGGTGGATGG<br>KP-MLST-phoE-R: GGTTAATCAGAACTGGTAGGTC	59℃
recA	347	KP-MLST-recA-F1: GCAATTCGGTAAAGGCTCCATC<br>KP-MLST-recA-R1: GAACATCACGCCAATTTTCATACG	59℃
rpoB	501	KP-MLST-rpoB-F: CGTCGTATCTCCGCACTCG<br>KP-MLST-rpoB-R: CGGGTACATCTCGTCTTCG	53℃

Note that the reference sequences of the former six loci are identical to those in the *K. pneumoniae* MLST Database at Institut Pasteur http://www.pasteur.fr/recherche/genopole/PF8/mlst/Kpneumoniae.html.

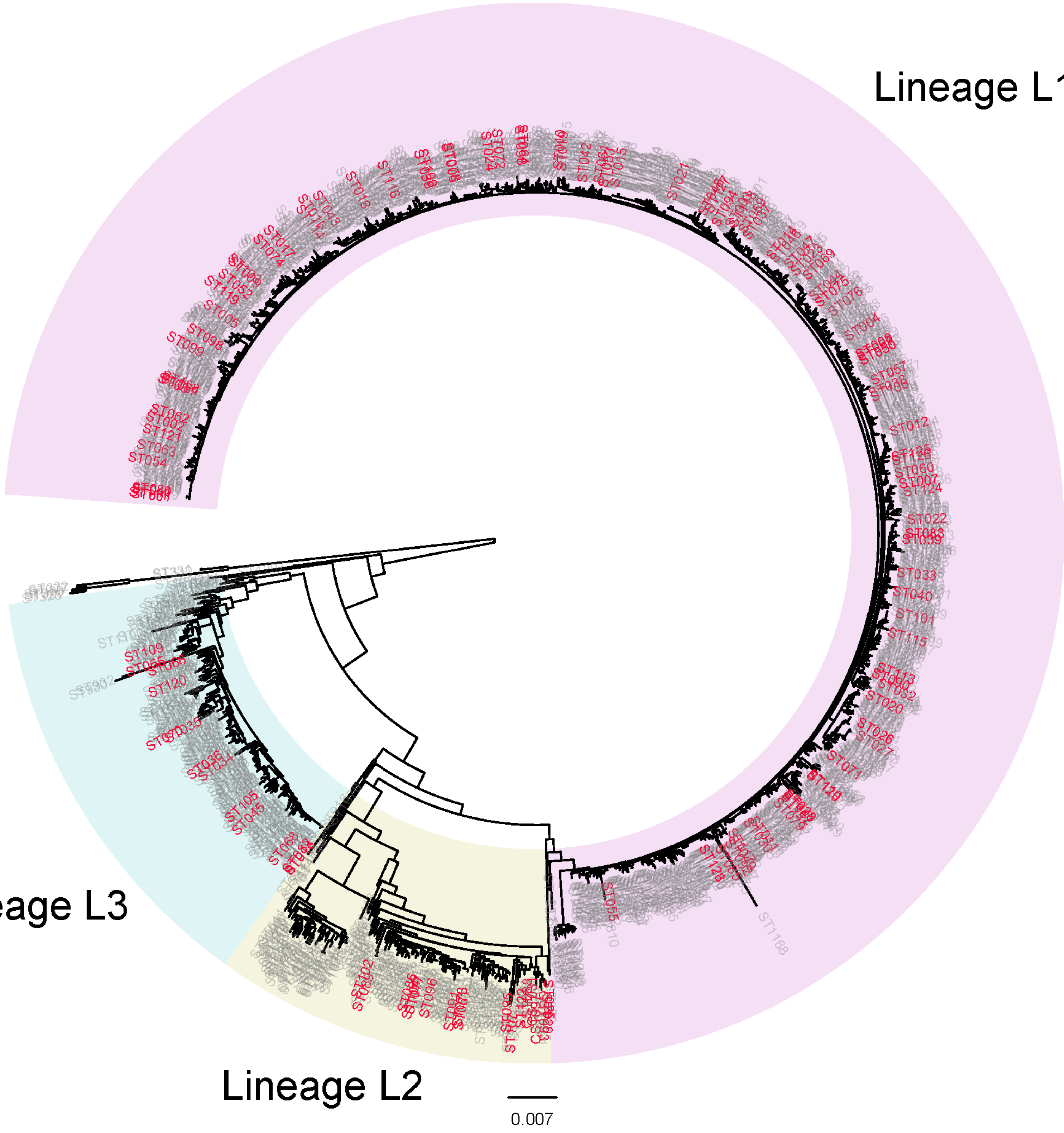

hylogenetic tree of 128 plus 1474 sequence types (STs). The unrooted NJ tree was generated from the concatenated sequences of the six loci (*gapA*, *infB*, *mdh*, *pgi*
STs (red) in this study and the 1474 STs (grey) in the *K. pneumoniae* MLST Database at Institut Pasteur (http://www.pasteur.fr/recherche/genopole/PF8/mlst/Kpneu
0, 2014). STs belonged to the three distinct lineages (see text for details) were denoted with different background colors.

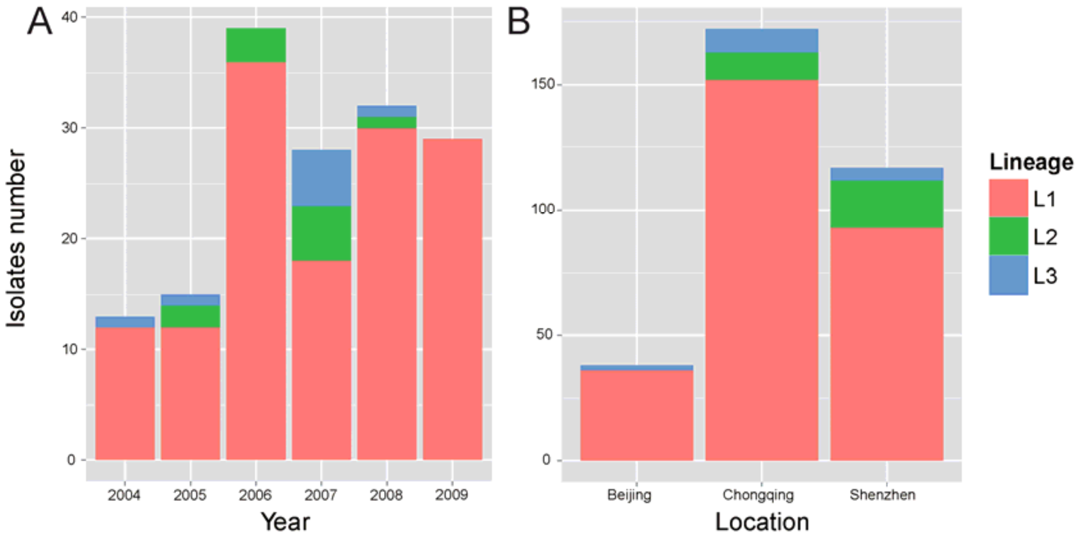

Figure S2 Time and geographical distribution of three lineages. A. Time distribution of 156/172 Chongqing isolates, which had isolating time and colored by lineages. B. Geographical distribution of all isolates colored by lineages.
